# The efficacy of tamsulosin in lower ureteral calculi

**DOI:** 10.4103/0974-7796.65110

**Published:** 2010

**Authors:** M.S. Griwan, Santosh Kumar Singh, Himanshu Paul, Devendra Singh Pawar, Manish Verma

**Affiliations:** Department of Surgery, Postgraduate Institute of Medical Sciences, Rohtak, Haryana, India; 1 Department of Urology, Postgraduate Institute of Medical Sciences, Rohtak, Haryana, India

**Keywords:** Tamsulosin, ureteral calculi, alpha blockers

## Abstract

**Context:**

There has been a paradigm shift in the management of ureteral calculi in the last decade with the introduction of new less invasive methods, such as ureterorenoscopy and extracorporeal shock wave lithotripsy (ESWL).

**Aims:**

Recent studies have reported excellent results with medical expulsive therapy (MET) for distal ureteral calculi, both in terms of stone expulsion and control of ureteral colic pain.

**Settings and Design:**

We conducted a comparative study in between watchful waiting and MET with tamsulosin.

**Materials and Methods:**

We conducted a comparative study in between watchful waiting (Group I) and MET with tamsulosin (Group II) in 60 patients, with a follow up of 28 days.

**Statistical Analysis:**

Independent 't' test and chi-square test.

**Results:**

Group II showed a statistically significant advantage in terms of the stone expulsion rate. The mean number of episodes of pain, mean days to stone expulsion and mean amount of analgesic dosage used were statistically significantly lower in Group II (*P* value is 0.007, 0.01 and 0.007, respectively) as compared to Group I.

**Conclusions:**

It is concluded that MET should be considered for uncomplicated distal ureteral calculi before ureteroscopy or extracorporeal lithotripsy. Tamsulosin has been found to increase and hasten stone expulsion rates, decrease acute attacks by acting as a spasmolytic, reduces mean days to stone expulsion and decreases analgesic dose usage.

## INTRODUCTION

Many minimally invasive interventional (e.g., ESWL and ureteroscopy) as well as expectant (watchful waiting) treatment exist for the management of lower ureteric calculi. But the choice of the ideal method to be taken up largely depend on the type of equipment available, type and size of stone, needs of the patient and the skills of the surgeon.[1] The stone burden remains the primary factor in deciding the appropriate treatment for a patient with ureteral calculi.[2] Where a failed expectant treatment may well be complicated with hydronephrosis, deranged renal function or urosepsis, interventional techniques are not always free of complications and failures.

Recent studies have reported excellent results relating to medical expulsive therapy (MET) for distal ureteral calculi, in terms of stone expulsion and control of ureteral colic pain, using drugs (e.g., nifedipine and prednisolone) that can modulate the function of the ureter obstructed by the stone. Recently, a a1A receptor blocker to be used in this regard is tamsulosin. Most of the work on the efficacy of tamsulosin in lower ureteral calculi expulsion has been done in western affluent countries with variable results. The disease spectrum in a developing country like ours, is different from that in developed countries, mainly because of delay in diagnosis, delay in investigations and lack of awareness which tend to modify the outcome in case of ureteral stones or for that matter any disease. More so, advanced interventional facilities in this part of the world are not easily available. A prospective study was thus planned to compare the tamsulosin group with a control group in our setup to evaluate the efficacy of tamsulosin for lower ureteral calculi expulsion within a few days without the need for hospitalization, common endoscopic treatment or shock wave lithotripsy.

## MATERIALS AND METHODS

A prospective randomized controlled study was conducted in the Department of Surgery and Urology, on OPD (outpatient department) basis. Sixty consecutive patients older than 18 years of age, presenting with a diagnosis of a symptomatic, unilateral, solitary lower ureteral stones (stone present at the level of ischial spine or below) proved either on a skiagram or sonography of the KUB (Kidney-Ureter-Bladder) with size ≥4 mm and ≤10 mm (in major axis) were included in this study.

All cases having active urinary tract infection, fever, acute renal failure, chronic renal failure, history of urinary surgery or endoscopic treatment, uncorrected distal obstruction and marked hydronephrosis were excluded from the study.

Prior to study, complete haemogram, blood urea, serum creatinine, urine complete examination, urine culture sensitivity, skiagram KUB after preparation and or sonography KUB were carried out on all patients enrolled for the study.

Total 60 symptomatic cases of lower ureteric stones were divided randomly into a control (group I) and a study group (group II).

Group I (Control) – The 30 patients included in this group was advised high fluid intake along with analgesic (tablet Diclofenac 50 mg)/spasmolytic (tablet hyoscine butylbromide 10mg) as on demand during the study period.

Group II (Study) – The 30 patients in this group were given Tab. Tamsulosin 0.4mg OD, in morning, half hour after breakfast for a maximum period of 28 days or till spontaneous passage of stone (which ever was earlier). High fluid intake and analgesic (tablet Diclofenac 50mg)/spasmolytic (tablet hyoscine butylbromide 10 mg) were given on demand during the study period.

The patients were followed up with a weekly sonography KUB and fortnightly X-ray KUB and final evaluation was done after completion of four weeks. Successful results were defined as complete stone passage and failure was considered if:

1. The patient failed to pass the stone at the end of 28 days.
2. Uncontrolled pain and/or uroseptic fever leading to hospitalization during study period.

## RESULTS

The study comprised of 60 patients. The youngest patient was 20 years of age while the oldest was 60 years of age. The mean age was 35.10 years.

The smallest stone was 4 mm in size while the largest stone was 10 mm in size. Majority of patients (81.66%) were having stones of size in the range of 5-8 mm. The mean stone size was 6.33 ± 1.47 (range 4–9) for Group I and 6.70 ± 1.60 (range 4–10) for Group II [Table 1].

**Table 1:** Stone size distribution

Size	Group I n (%)	Group II n (%)	Total n (%)
4 mm	3 (10)	2 (6)	5 (8)
5 mm	7 (23)	6 (20)	13 (22)
6 mm	7 (23)	7 (23)	14 (23)
7 mm	5 (17)	4 (13)	9 (15)
8 mm	6 (20)	7 (23)	13 (22)
9 mm	2 (6)	3 (10)	5 (8)
10 mm	0	1 (3)	1 (2)

*P*=0.359

There were 42 patients with right ureteral calculus and 18 with left ureteral calculus. There was an equal distribution of patients with right ureteral calculus and left ureteral calculus in both the groups [Table 1].

A stone expulsion rate of 70% (21 out of 30 patients) was observed for Group I and 90% (27 out of 30 patients) in Group II. Group II showed a statistically significant advantage in terms of the stone expulsion rate (*P*=0.04) as determined by chi-square test. The chi-square value for the test was 3.75 [Table 2].

**Table 2:** Data and results of randomization for mean days to expulsion of stones (mean expulsion time)

Expulsion time in days	Group I n (%)	Group II n (%)	Total n (%)
<7	8 (27)	15 (50)	23 (38)
7‐14	10 (33)	11 (37)	21 (35)
14‐21	3 (10)	1 (3)	4 (7)
21‐28	0	0	0
Stone not passed	9 (30)	3 (10)	12 (20)

χ^2^=6.18, *P*=0.01.

In Group I, 8 patients (27%) passed their stones within 7 days of treatment and 18 patients (60%) passed their stones within 14 days of treatment, while in Group II, 15 patients (50%) passed their stones within 7 days of treatment and 26 patients (87%) passed their stones within 14 days of treatment. As evident from chi-square test, Group II showed a statistically significant advantage in terms of expulsion time (in days) with a *p* value of 0.01 and chi-square value of 6.18 [Table 2].

The total number of patients with no episodes of pain during the study were 24, out of which 9 patients were in Group I while 15 patients were in Group II, showing significantly (*P*= 0.007) less number pain episodes in Group II as determined on the basis of independent 't' test.

Total 24 out of 60 patients did not use any analgesic medications during the trial. Only one patient required 200mg of diclofenac (each tablet 50mg) during a trial period of 28 days. Nine patients did not use any analgesics in Group I, while 15 patients did not used any analgesics in Group II. Mean amount of diclofenac sodium (in mg) was 63.33 ± 55.60 (range 0-200) per patient in Group I and 30.00 ± 33.73 (range 0–100) patients in Group II [Table 3].

**Table 3:** Summary of demographic data and results of randomization based on independent 't' test and chi-square test

	Group I (Control)	Group II (Study)	*P* value	Significance
Mean patient age (in yrs) ± SD (range)	36.00 ± 12.22 (20‐65)	34.20 ± 13.96 (20‐65)	0.597	Not significant
Mean stone size in mm ± SD (range)	6.33 ± 1.47 (4‐9)	6.70 ± 1.60 (4‐10)	0.359	Not significant
Stone expulsion rate in % (Number)	70 (21)	90 (27)	0.04	Significant
Mean no. of pain episode ± SD (Range)	1.27 ± 1.11 (0‐4)	0.60 ± 0.67 (0‐2)	0.007	Significant
Mean amount of analgesic dosage (in mg) ± SD (range)	63.33 ± 55.60 (0‐200)	30.00 ± 33.73 (0‐100)	0.007	Significant
Total number of patients	30	30	-	-
Stone site/localization (Right/Left)	21/9	21/9	-	-
Sex (Male/Female)	18/12	19/11	-	-
Emergency room visits	Nil	Nil	-	-
No. of hospitalization	Nil	Nil	-	-
Drug side effects	Nil	Nil	-	-

SD - Standard deviation

The mean number of episodes of pain, mean days to stone expulsion and mean amount of analgesic dosage used were statistically significantly lower in Group II (*P* value 0.007, 0.01 and 0.007, respectively) as compared to Group I [Table 3].

None of the patients underwent hospitalization or had emergency room visits [Table 3]. All the patients who were not stone free at the end of 28 days were successfully treated with ureteroscopy.

There was no statistically significant difference between the groups, with respect to age, sex, stone size and stone localization (right/left) in the present study and any other similar studies performed previously.

## DISCUSSION

Advances in endourological techniques and instrumentation have largely diverted the management of ureteral stones by open surgeries to either minimal invasive methods like ESWL and ureterorenoscopic removal of stones or to watchful waiting. The minimal invasive therapies for ureteral stone are now the accepted gold standards. Nevertheless, these techniques are not risk-free, are quite expensive[3] and are not widely available in the developing countries.

Watchful waiting is appropriate for small stones that are not causing acute symptoms and that are likely to pass spontaneously,[4] although it may occur at the expense of some discomfort to the patient. Spontaneous passage depends upon stone size, shape, location and associated ureteral edema (which is likely to depend on the length of time that a stone has not progressed). Ureteral calculi 4–5 mm in size have a 40–50% chance of spontaneous passage. In contrast, calculi >6 mm have less than 5% chance of spontaneous passage. Majority of stones that pass do so within a 6 weeks period after the onset of symptoms.[5] Smaller, more distal and right sided stones are more likely to pass spontaneously.[6,7] However, the expectant approach may result in complications, such as infection of the urinary tract, hydronephrosis and renal function defects.[7] In the present study the mean stone size (in mm) of Group I was 6.33 ± 1.47 with a range of 4–9 mm while it was 6.70 ± 1.60 in case of Group II with a range of 4–10 mm. The *P* value of mean stone size in mm amongst Group I and Group II was 0.359 (>0.05) and hence not significant with respect to stone passage.

α_1D_ receptors are found in abundance in the detrusor and the intramural part of the ureter. α_1A_ and α_1D_ adrenergic receptors are present more densely in the distal 1/3 of ureter (including intramural part) than other adrenergic receptors. When stimulated, they inhibit the basal tone, peristaltic wave frequency and the ureteral contractions even in the intramural part of lower ureter. α_1_ antagonists have a crucial impact in spontaneous painless elimination of the stones smaller than 8 mm located in the uretero-bladder junction.[8] They may work on the obstructed ureter by inducing an increase in the intraureteral pressure gradient around the stone, that is, an increase in the urine bolus above the stone (and consequently an increase in intraureteral pressure above the stone) as well as decreased peristalsis below the ureter (and consequently a decrease in intraureteral pressure below the stone) in association with the decrease in basal and micturition pressures even at the bladder neck, thereby an increased chance of stone expulsion. Furthermore, the decreased frequency of phasic peristaltic contractions in the obstructed ureteral tract induced by tamsulosin might determine a decrease in or the absence of the algogenic stimulus.[9]

Cervenakov *et al*, concluded that the treatment by α_1_ blockers considerably decreased not only lower urinary tract symptoms (LUTS) but also helped to accelerate the passing of minor calculi from the terminal parts of the ureters of 80.4% of patients. They also suggested that α_1_ blockers potentiate the spasmoanalgesic action of drugs used in standard methods of treatment.[10] In the present study, the mean amount of analgesic dosage (in mg) was 63.33 ± 55.60 (range 0–200) in Group I, while the amount was 30.00 ± 33.73 (range 0–100) in Group II with a *P* value=0.007 (statistically significant).

Dellabella *et al*, used tamsulosin as a spasmolytic drug during episodes of ureteral colic due to juxtavesical calculi, observed an increased stone expulsion rate and with a decrease in stone expulsion time, the need for hospitalization and endoscopic procedures, and provided particularly good control of colic pain.[9] Addition of tamsulosin to conventional treatment is beneficial in terms of clearance of lower ureteral stones and this effect was more evident for larger stones, especially when combined with shock wave lithotripsy (SWL).[11] In the present study, the patients in Group I had a mean number of 1.27 episodes of pain with a range of 0–4, while in Group II, the mean number of pain episodes were 0.60 with a range of 0–2 (statistically significant, *P*=0.007).

Corticosteroid drug in association with tamsulosin seemed to induce more rapid stone expulsion. In addition, tamsulosin alone as MET for distal ureteral calculi had excellent expulsive effectiveness.[12]

Alfa1-blockers decreased the number of ureteral colic episodes and the intensity of pain during spontaneous passage at the lower ureteral calculi. Also, it was beneficial to patients' quality of life.[13]

## CONCLUSION

It is concluded that MET should be considered for uncomplicated distal ureteral calculi before ureteroscopy or extracorporeal lithotripsy. Tamsulosin has been found to increase and hasten stone expulsion rates, decrease acute attacks by acting as a spasmolytic, reduces mean days to stone expulsion and decreases analgesic dose usage. Appropriately used it may have substantial fiscal benefits by reducing the number of interventional procedures and the acute attacks too. However, this requires larger prospective randomized controlled trials before its application can be universally recommended.
